# Algorithm selection for protein–ligand docking: strategies and analysis on ACE

**DOI:** 10.1038/s41598-023-35132-5

**Published:** 2023-05-22

**Authors:** Tianlai Chen, Xiwen Shu, Huiyuan Zhou, Floyd A. Beckford, Mustafa Misir

**Affiliations:** Department of Natural and Applied Sciences, Duke Kunshan University, Kunshan, China

**Keywords:** Computational biology and bioinformatics, Chemistry, Mathematics and computing

## Abstract

The present study investigates the use of algorithm selection for automatically choosing an algorithm for any given protein–ligand docking task. In drug discovery and design process, conceptualizing protein–ligand binding is a major problem. Targeting this problem through computational methods is beneficial in order to substantially reduce the resource and time requirements for the overall drug development process. One way of addressing protein–ligand docking is to model it as a search and optimization problem. There have been a variety of algorithmic solutions in this respect. However, there is no ultimate algorithm that can efficiently tackle this problem, both in terms of protein–ligand docking quality and speed. This argument motivates devising new algorithms, tailored to the particular protein–ligand docking scenarios. To this end, this paper reports a machine learning-based approach for improved and robust docking performance. The proposed set-up is fully automated, operating without any expert opinion or involvement both on the problem and algorithm aspects. As a case study, an empirical analysis was performed on a well-known protein, Human Angiotensin-Converting Enzyme (ACE), with 1428 ligands. For general applicability, AutoDock 4.2 was used as the docking platform. The candidate algorithms are also taken from AutoDock 4.2. Twenty-eight distinctly configured Lamarckian-Genetic Algorithm (LGA) are chosen to build an algorithm set. ALORS which is a recommender system-based algorithm selection system was preferred for automating the selection from those LGA variants on a per-instance basis. For realizing this selection automation, molecular descriptors and substructure fingerprints were employed as the features characterizing each target protein–ligand docking instance. The computational results revealed that algorithm selection outperforms all those candidate algorithms. Further assessment is reported on the algorithms space, discussing the contributions of LGA’s parameters. As it pertains to protein–ligand docking, the contributions of the aforementioned features are examined, which shed light on the critical features affecting the docking performance.

## Introduction

In the wake of emerging diseases and rising awareness of the desire to improve human well-being, there has been a persistent effort to implement new medical innovations. A broad array of concepts in Drug Discovery/Design (DD)^1^ has been the leading topics of interest. The DD process, however, is time-consuming and expensive. The entire DD pipeline can last as long as 15 years, requiring high budgets and the participation of large groups of scientists. In that respect, the traditional DD process often comes with a high cost and risk and a low success rate, factors that discourage new research and hinder substantive advances in this field^2^. A major factor that contributes to this fact is that DD is essentially a search problem of the enormous chemical space to detect druggable compounds^3,4^. Arguably, the most critical step in this arduous process is identifying the new chemical compounds that could be developed into new medicines.

Computational approaches have been practical, in general, as they are effective mechanisms to move the DD process forward at an increased pace, with improved successful outcomes. Computer-Aided DD (CADD)^5–10^ is an umbrella term covering those computational procedures. To be specific, CADD is a collection of mathematical and data-driven tools that cut across disciplines with respect to their utilization in DD. These tools are implemented as computer programs and are accommodated in conjunction with varying experimental methodologies to expedite the discovery of new chemical entities. The CADD strategies can quickly triage a very large number of compounds, identifying hits that can be converted to leads. The laboratory methods then take over for testing and finalizing the drug. This process is iterative and reciprocal. The outcomes of the CADD methods are exploited to devise compounds that are subjected to chemical synthesis and biological assay. The information derived from those experiments is exploited to further develop the structure activity relationships (SARs) and quantitative SARs (QSARs) that are embedded in the CADD approaches.

Among the CADD methods, molecular docking has been particularly popular. Molecular docking is the process by which a small molecule, generally referred to as a ligand, is computationally interacted with a protein or other biomolecules without any laboratory work. Procedurally, it varies the ligand’s conformation and orientation in limited and stochastic steps. Its goal is to seek the best docking conformation, or pose, that minimizes the binding energy. The results returned by the molecular docking programs are usually the binding energy value and a protein–ligand complex file that are indicative of the actual binding affinity and position when the ligand is co-crystalized with the receptor. Molecular docking has been benefited in different CADD procedures, including virtual screening, a process that queries the binding of a large number of molecules to a particular disease (biological) target.

This study aimed at applying Algorithm Selection (AS)^11,12^ to automatically suggest algorithms that best solve the Protein–Ligand Docking problem (PLDP). The idea of AS is motivated by the No Free Lunch Theorem (NFLT)^13^. The NFLT essentially states that every algorithm performs the same on average when it is applied to all possible problem instances. Thus, every algorithm has its own strengths and weaknesses, no matter how complex and advanced it is. AS basically attempts to choose the most suitable algorithm from an existing pool of algorithms to address a given problem instance of any domain. The objective of this work was to identify the most suitable algorithm from a fixed pool of PLDP algorithms for each given PLDP instance. AutoDock4^14^ was preferred as it is a widely used PLDP tool, supplying a favorable algorithm pool. An existing AutoDock solver, Lamarckian GA (LGA)^15^, which integrates the Genetic Algorithm (GA)^7^ and the Local Search (LS)^16^, was used in a parameterized manner such that a suite of candidate algorithms was derived. This step resulted in 28 LGA variants, including the LGA with its default parameter values. They were used on 1428 PLDP instances, each concerning one ligand out of 1428 ligands and a single target protein of Human Angiotensin-Converting Enzyme (ACE). Those 28 algorithms are managed by ALORS^17^, which is a recommender systems-based AS approach. To be able to use AS, a feature set is derived for representing the PLDP instances, including the widely adopted molecular descriptors as well as the substructure fingerprints. Following this setup, an in-depth experimental analysis is reported, initially comparing each standalone LGA variant against ALORS. Concerning the analysis capabilities of ALORS, the resemblance of the candidate algorithms—in terms of the LGA parameter values in this case—and the PLDP instance similarities besides the importance of the LGA parameters and PLDP instance features are investigated. The consequent assessment provides practical insights for how to use LGA with increased performance and what to consider when solving a particular PLDP scenario.In the remainder of the paper, Section "Methods" discusses the relevant literature both on PLDP and AS after formally describing them. The AS method employed for choosing algorithms is detailed in Section "Results and discussion". A comprehensive computational analysis and discussion are provided in Section "Conclusion".

### Protein ligand docking

Protein–ligand docking plays a crucial role in modern pharmaceutical research and drug development. Docking algorithms estimate the structure of the ligand-receptor complex through sampling and ranking. They first sample the conformation of the ligands in the active site of a receptor. Next, they rank all the generated poses based on specific scoring functions or simply by calculating the binding energy^18^. Docking algorithms are thus capable of simulating the best orientation of a ligand when it is bound to a protein receptor.

The initial docking technique is based on the Fischer’s lock-and-key assumption^19^. This assumption treats both the ligand and the receptor as rigid bodies with their affinity proportional to their geometric forms. In most elementary rigid-body systems, the ligand is sought in a six-dimensional rotational or translational space to fit the binding site. Later, Koshland proposed the theory induced-fit^20^, which implies that ligand interactions would continuously modify the active site of a receptor. In essence, the docking procedure is considered dynamic and adoptable. In the last several decades, numerous docking technologies and tools have been developed, such as DOCK^21^, AutoDock^22^, GOLD^23^, and Glide^24^. Besides the differences in the implementation of 3D pose investigation, protein receptor modeling, etc., the major variation among them is the evaluation of the binding affinity, performed by different Scoring Functions (SFs)^25^. The existing scoring functions can be categorized as (1) force field based, (2) empirical function based, and (3) knowledge based^26^. Because of the heterogeneity of how protein–ligand interaction is modeled in different scoring functions, it is likely that diverse performance can be observed if one scoring function is applied to all docking tasks.

This study utilized AutoDock4 as it is an open-source, widely used system. It is the first docking software that can model ligands with complete flexibility^27^. AutoDock4 consists of two fundamental software components: AutoDock and AutoGrid. While AutoDock is the main software, AutoGrid calculates the noncovalent energy of interactions and produces an electrostatic potential grid map^28^. As a feature of AutoDock4^27^, it is possible to model receptor flexibility by shifting side chains. To deal with side-chain flexibility, a simultaneous sampling method is provided. While the other chains stay stiff, the user-selected chains are sampled by a certain method with the ligand. With AutoGrid, the rigid portion is processed as a grid energy map. The grid maps together with the receptor’s flexible portion direct the selected ligands’ docking process^28^.

AutoDock4 adopts the physics-based force field scoring function with van der Waals, electrostatic, and directional hydrogen-bond potentials derived from an early version of the AMBER force field^29^. In addition, a pairwise-additive desolvation term based on partial charges, and a simple conformational entropy penalty are included^26^. The scoring function consists of electrostatic and Lennard–Jones VDW terms:

$$ E = \mathop \sum \limits_{i} \mathop \sum \limits_{j} \left( {\frac{{A_{ij} }}{{r_{ij}^{12} }} - \frac{{B_{ij} }}{{r_{ij}^{6} }} + \frac{{q_{i} q_{j} }}{{{\upvarepsilon }\left( {r_{ij} } \right)r_{ij} }}} \right) $$

where $$A_{ij}$$ and $$B_{ij}$$ are the VDW parameters, $$r_{ij}$$ refers to the distance between the protein atom $$i $$ and the ligand atom $$j $$, and $$q_{i}$$ and $$q_{j}$$ are atomic charges. $$\varepsilon \left( {r_{ij} } \right)$$ is introduced as a simple distance-dependent dielectric constant in the Coulombic term. However, the desolvation effect cannot be represented in the Coulombic term^26^. The ignored solvent effect will lead to a biased scoring function that will not consider those relatively low-charged ligands.

A knowledge-based scoring function^25^ is further established based on the statistical mechanics of interacting atom pairs. A pairwise additive desolvation term is introduced, which is directly obtained from the frequency of occurrence of atom pairs by the Boltzmann relation. The energy potentials derived from structural information are also included in determining atomic structures^26^. The potentials are calculated by

$$ w\left( r \right) = - {\upkappa }_{B} T\log \left[ {g\left( r \right)} \right],g\left( r \right) = \frac{\rho \left( r \right)}{{{\uprho }*\left( r \right)}} $$

where $$\kappa_{B}$$ is the Boltzmann constant, $$T $$ is the absolute temperature of the system, $$\rho \left( r \right)$$ is the number density of the protein–ligand atom pair at distance $$r $$, and $$\rho *\left( r \right)$$ is the pair density when interatomic interactions are zero. The inverse Boltzmann stands for the mean-force potentials, not the true potentials, which are quite different from the simple fluid system^26^. Thus, although it excludes the effects of volume, composition, etc., it still helps to convert the atom–atom distances into a function suitable for complex protein systems.

Most AutoDock4 users, as well as users of other molecular docking platforms, tend to follow the recommended docking protocol with the given default values. This practice is mainly followed to avoid tweaking the docking program. Furthermore, some docking programs including AutoDock4, only provide a limited set of options for executing the search with a particular scoring function, but there still remain a lot of other combinations. In the case of AutoDock4, the recommended choice of algorithm is the Lamarckian Genetic Algorithm (LGA). That being said it is possible to show docking scenarios where LGA performs relatively poor.

### Algorithm selection

The selection of appropriate algorithms for problem solving in a variety of contexts has drawn increasing attention in the last few decades^30^. A phenomenon known as performance complementarity argues, based on empirical research, that one algorithm may perform well in one setting while others perform better in other conditions^12^.

The concept of per-instance algorithm selection was proposed and examined^11^. This idea refers to finding which algorithm is the best for a given instance^12^. The rationale for the in-depth examination of this algorithm is the selection of a suitable algorithm from a huge number of diverse existing algorithms. However, it took decades to become widespread for being applied to address Boolean satisfiability (SAT) and other difficult combinatorial problems^31^. In the designated procedure, a rule is developed between an appropriate algorithm and a certain scenario. In optimization issues, per-instance algorithm selection has therefore become prominent.

As the application of machine learning methods has been proven to be competent in many tasks, an automatic rule-connecting method has been studied^12^. Detailed and insightful instructions^32^ have been provided on the first automatic algorithm selection process and it has addressed a number of important issues, including the selection of regression or classification and the distinction between dynamic and static feature. However, continuous issues have been omitted. Furthermore, a generalization to the continuous optimization problem^33^ has been proposed by highlighting the benefits of discrete problems.

## Methods

**Figure 1:**
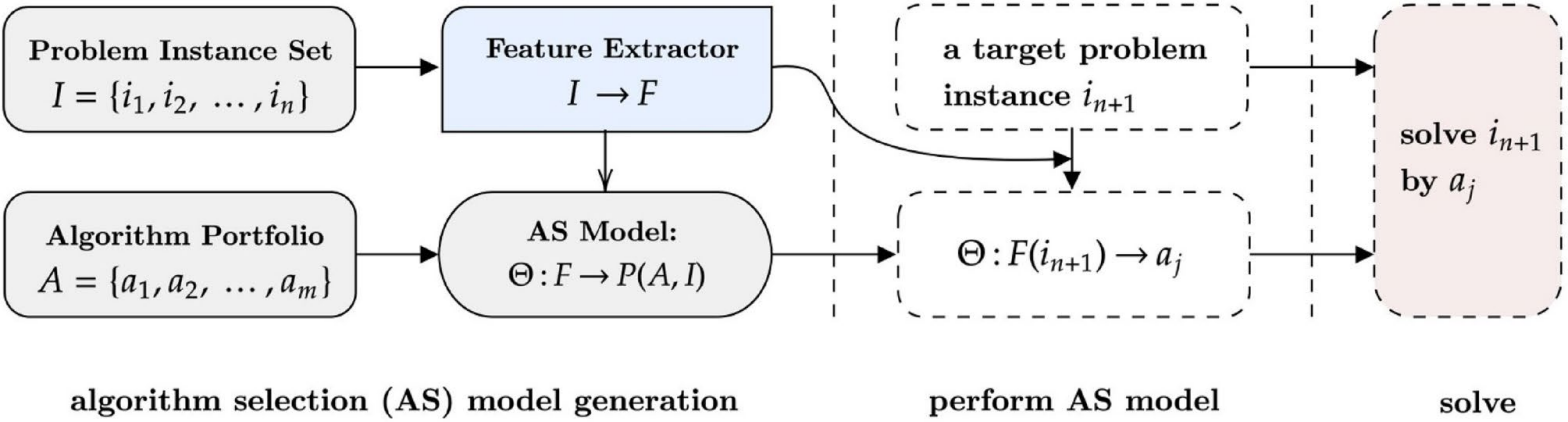
Illustration of Algorithm Selection. The traditional per instance Algorithm Selection (AS) process.

The main component of the proposed approach is the algorithm selection (AS) module as visualized in Fig. 1. It is responsible for choosing an algorithm in a per-instance manner and for matching a suitable algorithm to address a given (PLDP) instance. Also, referring to the earlier AS description, initially a group of PLDP algorithms, A, should be provided. Although these algorithms can be determined and used in a fixed way, algorithm portfolio generation strategies^34–36^ can be incorporated to derive candidate algorithms. Alongside an algorithm set, an instance set $I$, should be accommodated to model the AS system. Although AS is a problem-independent strategy, the behavior of AS is highly affected by the choice of those instances. If the AS is planned to be used to realize a rather specific family of docking tasks, $\mathcal{I}$ can include the instances from that particular family. Otherwise, to have a generalized AS model, it is beneficial for $I$ to contain a wide range of diverse PLDP instances. In the current study, there is only one target protein, yet a rather large set of ligands. Thus, any built AS model here is specific to that target protein while having some level of generality regarding the ligands. In relation to this diversity aspect, having a high diversity through complementarity in $\mathcal{A}$ can potentially offer improved and robust AS models. The complementarity, here, denotes having algorithms with varying problem solving capabilities. While an algorithm works well on a certain type of instance, another algorithm can perform well on instances where the earlier algorithms perform poorly. The chosen $A$ and $I$ are then used to generate performance data, $P(A, I)$, denoting the performance of each candidate algorithm, $a$, on each problem instance, $P(a, i) = {p}_{ai}$. During this performance data generation step, it is critical to take into account the stochastic / non-deterministic nature of candidate algorithms. This means that if an algorithm may deliver a different solution after each run on the exact same problem instance, it will be misleading to run that algorithm only once and use that value in $P$. In such cases, it is reasonable to run those algorithms multiple times and use their mean or median values as their per-instance performance indicators. One last element required to build an AS model is to specify the number of features, $F$, adequately describing the characteristics of the target problem instances. With data manipulation or data format conversions, this step can be skipped as the features are automatically derived^37^. Otherwise, with the help of the chemistry experts, reasonably representative instance features can be collected. Yet, it is potentially possible to come up with such features referring to the relevant literature, without the need of the actual presence of experts. That said, depending on the target problem, it might be good enough to solely utilize basic statistical measures and values achieved via landmarking^38^. At this point, traditionally, an AS model can be built, in the form of performance prediction, $\Theta :F\left(I\right)\to P\left(A,I\right)$, or other existing AS strategies can be employed.

**Figure 2:**
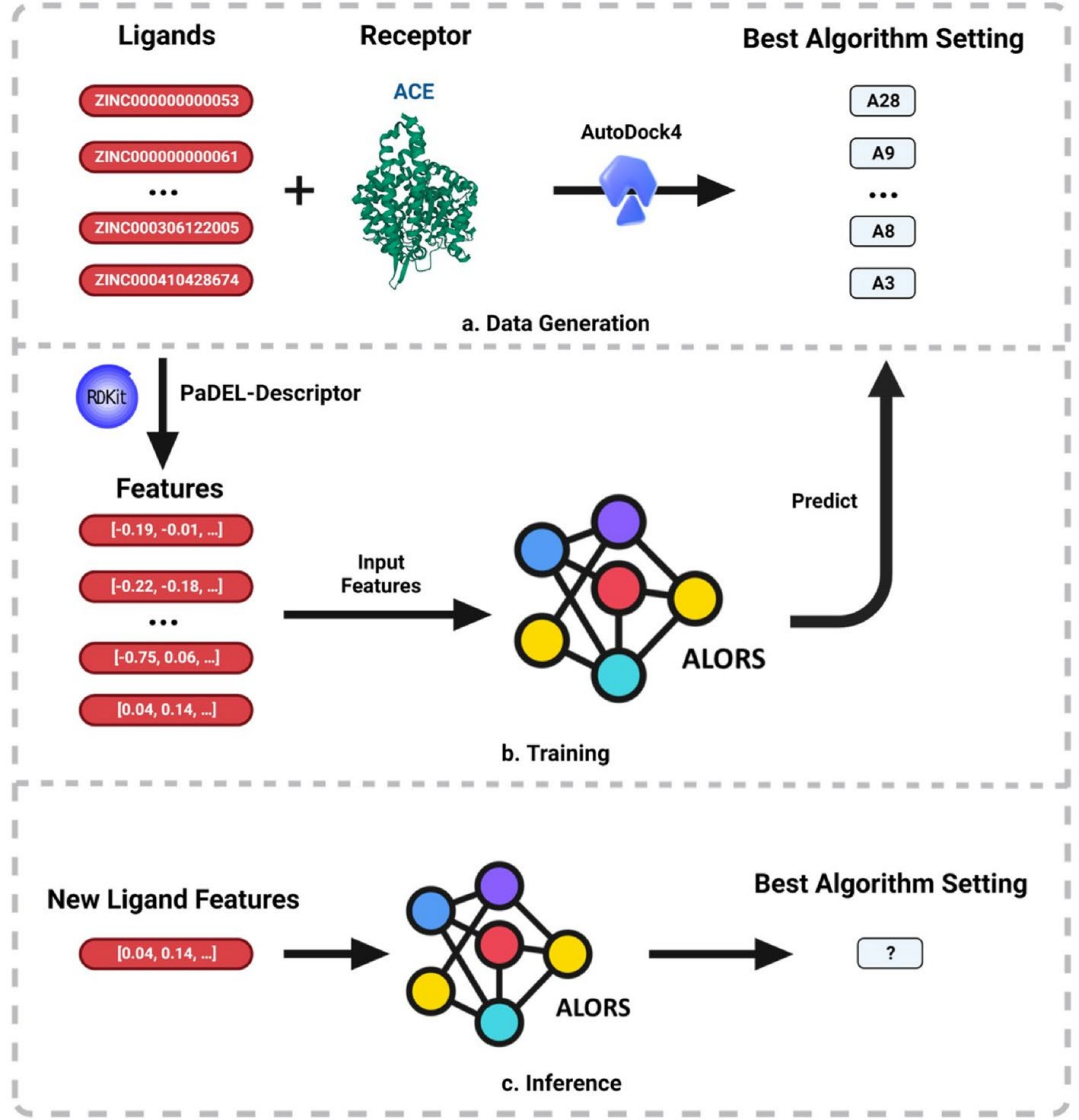
Framework of ALORS for Protein–Ligand Docking. All ligands are docked with ACE using 28 algorithms, each with a different parameter configuration in AutoDock4 during the data generation procedure. The algorithm configuration that produces the lowest docking scores averaged for 50 runs is selected as the best algorithm for the given instance, such as the 28th algorithm setting (A28). The ALORS model is trained using molecular descriptors and fingerprints, and the best algorithm labels corresponding to each ligand. Our model uses features of a single new ligand to determine the best algorithm configuration for inference.


Following the given framework, Fig. 2 visualizes the AS setting performed in this article. The data generation step is achieved based on AutoDock 4.2. For the AS method, an existing technique, ALORS1^17^, is recruited. ALORS is an algorithm recommendation system, based on collaborative filtering (CF)^39^. It has been successfully applied for different selection decisions on varying problem domains^40–43^, including those on a relevant protein-structure prediction problem^44,45^. CF is a type of recommendation approach, that predicts how much users like certain items such as movies and products. It makes predictions based on relating similar entries both at the user and item levels. Unlike other recommendation methods, CF works with sparse entries. ALORS accommodates the CF idea by considering problem instances as the users while considering algorithms as the items; that is, how much an instance likes an algorithm, depending on the relative success of the algorithm compared to all the candidate algorithms. Similar to the CF applications, ALORS also works with rank-based data, the ranks of all the present algorithms on all the problem instances. In that respect, ALORS performs algorithm selection (AS) as a rank-prediction task. However, unlike the existing AS systems, ALORS indirectly performs rank predictions. Essentially, a prediction model derived by ALORS is a feature-to-feature model, as detailed in Algorithm 1. It maps a set of hand-picked features characterizing the target problem instances to another group of instance features. The latter suite of features is the ones automatically extracted from the rank performance data by Matrix Factorization (MF). To be specific, Singular Value Decomposition (SVD)^46^ is used as the MF method for dimensionality reduction.

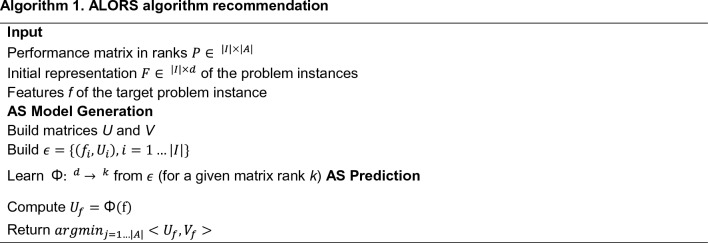


ALORS here is applied with k = 5 with respect to the rank of MF by SVD. Regarding to the modelling component of Random Forest (RF)^47^, the number of trees is set to 100 which is the default value in Scikit.

The candidate algorithm set is composed of 28 algorithms while the number of docking scenarios, instances, is 1428. The algorithms are essentially specified by setting distinct parameter configurations of a Lamarckian- Genetic Algorithm (LGA), as detailed in Table 1. The evaluation is realized through tenfold cross-validation (10-cv).

**Table 1:** Docking configurations.

Algorithm	Population size	Mutation rate	Window size
A1	50	0.02	10
A2	150	0.02	10
A3	200	0.02	10
A4	150	0.5	10
A5	150	0.8	10
A6	150	0.02	30
A7	150	0.02	50
A8	50	0.02	30
A9	50	0.02	50
A10	200	0.02	30
A11	50	0.5	10
A12	50	0.5	30
A13	50	0.5	50
A14	50	0.8	10
A15	50	0.8	30
A16	50	0.8	50
A17	150	0.5	30
A18	150	0.5	50
A19	150	0.8	30
A20	150	0.8	50
A21	200	0.02	50
A22	200	0.5	10
A23	200	0.5	30
A24	200	0.5	50
A25	200	0.8	10
A26	200	0.8	30
A27	200	0.8	50
A28	150	0.02	10

The standalone docking algorithms are derived from a Lamarckian-Genetic Algorithm (LGA). A28 use classical Solis and Wets local searcher (sw), and the rest use pseudo-Solis and Wets local searcher (psw). Population size: the number of individuals, i.e., solutions, maintained in each generation of LGA. A larger population size typically requires more computational resources as more searches are performed before convergence. Mutation rate: the probability that an individual is mutated, i.e., a solution is manipulated. A higher mutation rate will lead to more exploratory searches while a lower rate renders the search more exploitative. Window size: the size of the energy window that is used to determine which individuals will be subjected to the local search procedure. A smaller window size will result in more focused refinement of the best individuals, while a larger one keeps more individuals for the local search.

The ligands are molecules approved by the U.S. Food and Drug Administration (FDA) 2 in ZINC15 database^48^. Human Angiotensin Converting Enzyme (ACE), a critical membrane protein for the SARS-COV virus, and renal and cardiovascular function, is chosen as the target receptor (PDB DOI: 1O86)^49^. The original ligand files are in MOL2 format and are converted to PDB format for docking via Openbabel^50^. Receptors and ligands are preprocessed by AutoDock Tools and include addition of hydrogen bonds and charges in the form of PDBQT. The whole docking process is performed via AutoDock 4.2. The random seed is fixed for the repeatability of the experiment. Each algorithm is set to run for 50 times for each ligand and the number of energy evaluations is set to 2, 500, 000. They are both fixed to control the computational resources each algorithm can utilize. The rest of the settings are default with details described in AutoDock4’s user guide 3. For feature extraction, RDKit^51^ is used to generate molecular descriptors, and the PubChem Substructure Fingerprints are computed by PaDEL-Descriptor^52^. Molecular descriptor are the numerical values of a molecule’s properties computed by algorithms^51^. After the removal of the descriptors with the value 0 across all ligands, 208 features are obtained. Following this step, the features with almost the same values across different ligands are discarded, which results in 119 useable features. All the features are determined through min–max normalization, fitting each feature’s values to [0, 1]. PubChem Substructure Fingerprint is an ordered list of binary values (0/1), which represents the existence of a specific substructure, such as a ring structure^53^. In our case, for each ligand, the length of binary encoded list is 881.

## Results and discussion

**Figure 3:**
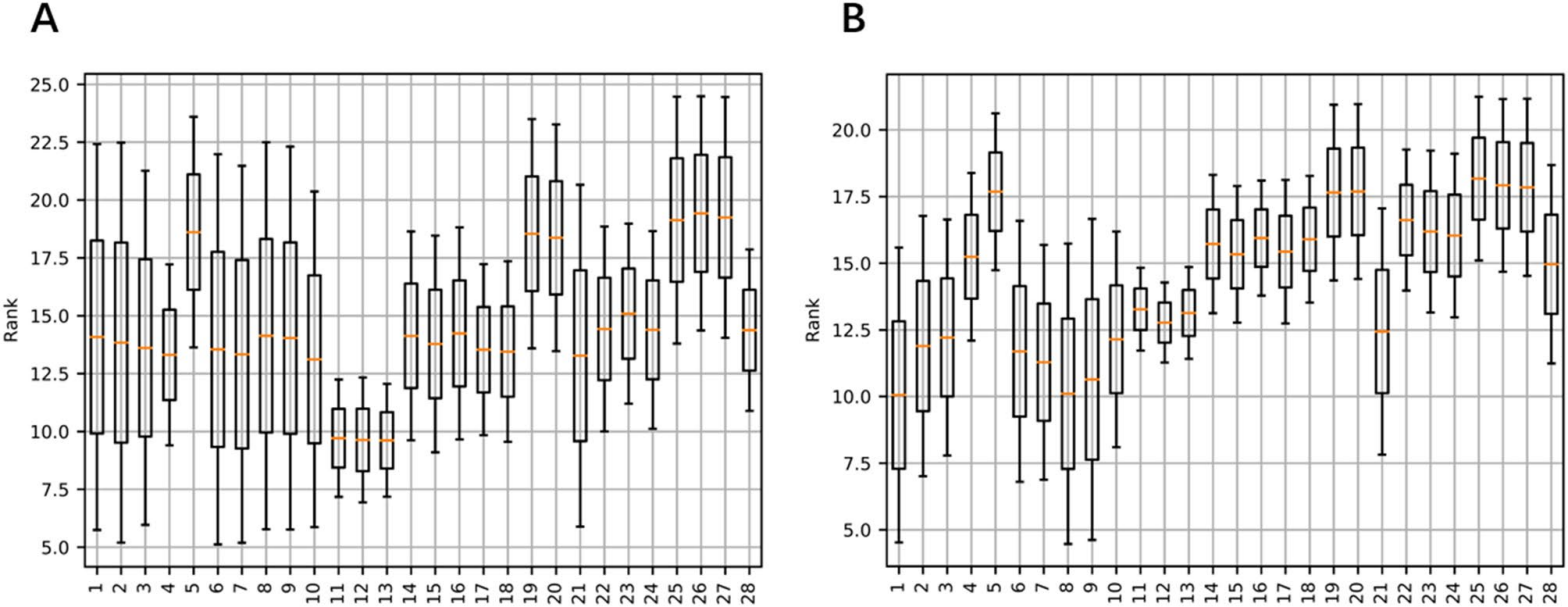
Ranks of Docking Algorithms. (**A**) The ranks of the docking algorithms across all the instances, based on the AVG performance. (**B**) The ranks of the docking algorithms across all the instances, based on the BEST performance.

Figure 3 illustrates the ranks of each algorithm across all the docking scenarios for AVG and BEST, respectively. It can be seen that while some algorithms perform better than other in general, their relative performances vary. Beyond that, there is no ultimate algorithm that consistently outperforms the remaining algorithms on all the protein–ligand docking instances. This view suggests that algorithm selection is likely.to beat all these algorithms by automatically matching the right algorithms with the instances that can be effectively solved by the selected algorithms.

Table 2 reports the ranking of each standalone algorithm besides ALORS. All those algorithms are accommodated as the candidate algorithms for ALORS. Two separate performance evaluation is delivered. The first one focuses on the algorithms’ average performance, considering that all the utilized algorithms are stochastic. The second case relates to the best docking solutions out of all the runs on each docking instance. For both scenarios, ALORS outperforms all the standalone algorithms, while the performance difference on the AVG case is more drastic than in the BEST case.

**Table 2:** Algorithm performance.

Algorithm	AVG Perf	BEST Perf
	Mean ± SD	Mean ± SD
A1	8.48 ± 8.00	6.82 ± 6.40
*A2*	**8.04** ± **6.82**	**9.92** ± **6.22**
A3	8.59 ± 6.41	9.96 ± 5.95
A4	17.13 ± 5.03	18.22 ± 6.70
A5	23.25 ± 5.25	20.55 ± 6.71
***A6***	**7.90** ± **6.79**	9.09 ± 5.88
A7	7.91 ± 6.62	8.99 ± 5.89
***A8***	8.50 ± 8.17	**6.80** ± **6.31**
A9	8.48 ± 8.07	7.09 ± 6.65
A10	8.38 ± 6.15	10.16 ± 5.89
A11	12.47 ± 4.27	15.16 ± 6.28
A12	12.50 ± 4.19	14.61 ± 6.20
A13	12.29 ± 4.31	15.12 ± 6.35
A14	18.40 ± 5.46	18.33 ± 6.83
A15	18.17 ± 5.45	17.92 ± 6.87
A16	18.56 ± 5.52	18.26 ± 6.70
A17	17.20 ± 4.98	18.12 ± 6.63
A18	17.27 ± 5.08	18.37 ± 6.56
A19	23.16 ± 5.29	20.77 ± 6.65
A20	22.94 ± 5.35	20.80 ± 6.73
A21	8.45 ± 6.24	10.05 ± 6.18
A22	18.63 ± 5.35	19.28 ± 6.51
A23	18.91 ± 5.17	19.12 ± 6.64
A24	18.49 ± 5.29	18.98 ± 6.73
A25	24.04 ± 5.33	21.14 ± 6.71
A26	24.12 ± 5.16	21.00 ± 6.78
A27	24.05 ± 5.44	20.98 ± 6.80
A28	12.66 ± 6.05	13.29 ± 6.77
ALORS	6.00 ± 5.14	6.75 ± 5.90

The ranking of the protein–igand docking algorithms with ALORS, utilizing all those standalone algorithms as a high-level approach. (The results in bold refer to the overall best ones).

Overall, ALORS consistently delivers the top and most robust performance across all docking instances. The robustness aspect can be verified from the standard deviation values. Taking a closer look at the results and referring to the AVG performances, A6 happens to be the best standalone algorithm, meaning that it is traditionally used as the sole algorithm for all the docking instances, unlike AS, choosing one docking algorithm for each docking instance. While A6’s mean rank is 7.90, ALORS results in the mean rank of 6.00. A6 is followed by A7, with a mean rank of 7.91. Additionally, the default algorithm setting that is built into AutoDock, A2, is found to be the third best standalone approach on the present test scenarios. As to delivering the BEST docking results, unlike the AVG case, A8 offers the top mean rank of 6.80, among the constituent algorithms, following ALORS’ mean rank of 6.75. A1 offers a performance quite close to A8, with a mean rank of 6.82. The closest performer after A1 is A9 with the mean rank of 7.09. The default configuration of A2 takes the fifth place among these standalone methods.

**Figure 4:**
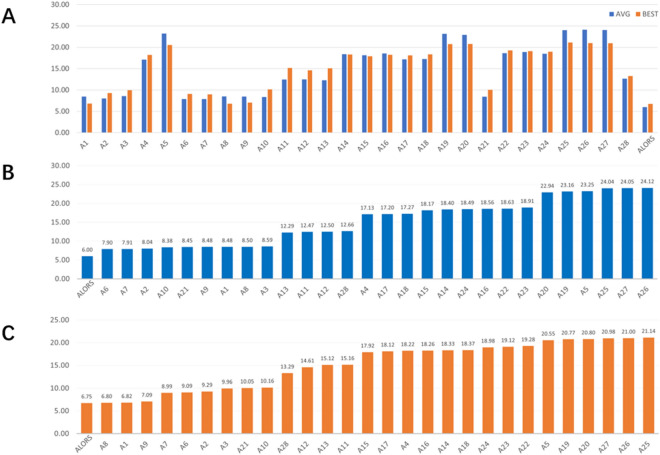
Mean Ranks of Docking Algorithms. The mean ranks of all the tested docking methods. (**A**) relative comparison on both AVG and BEST, (**B**) sorted comparison on AVG, (**C**) sorted comparison on BEST.

Figure 4 visualizes the mean rank changes for both AVG and BEST, referring to the top chart. It is noteworthy that the relative performance trend among all the algorithms is somewhat maintained. The remaining charts shows the sorted docking methods on AVG and BEST, separately. Just by visually analyzing the charts, closely ranked methods, in groups, can be detected. For instance, A5, A19, A20, A25, A26, and A27 clearly deliver the worst performance among all algorithms.

**Figure 5:**
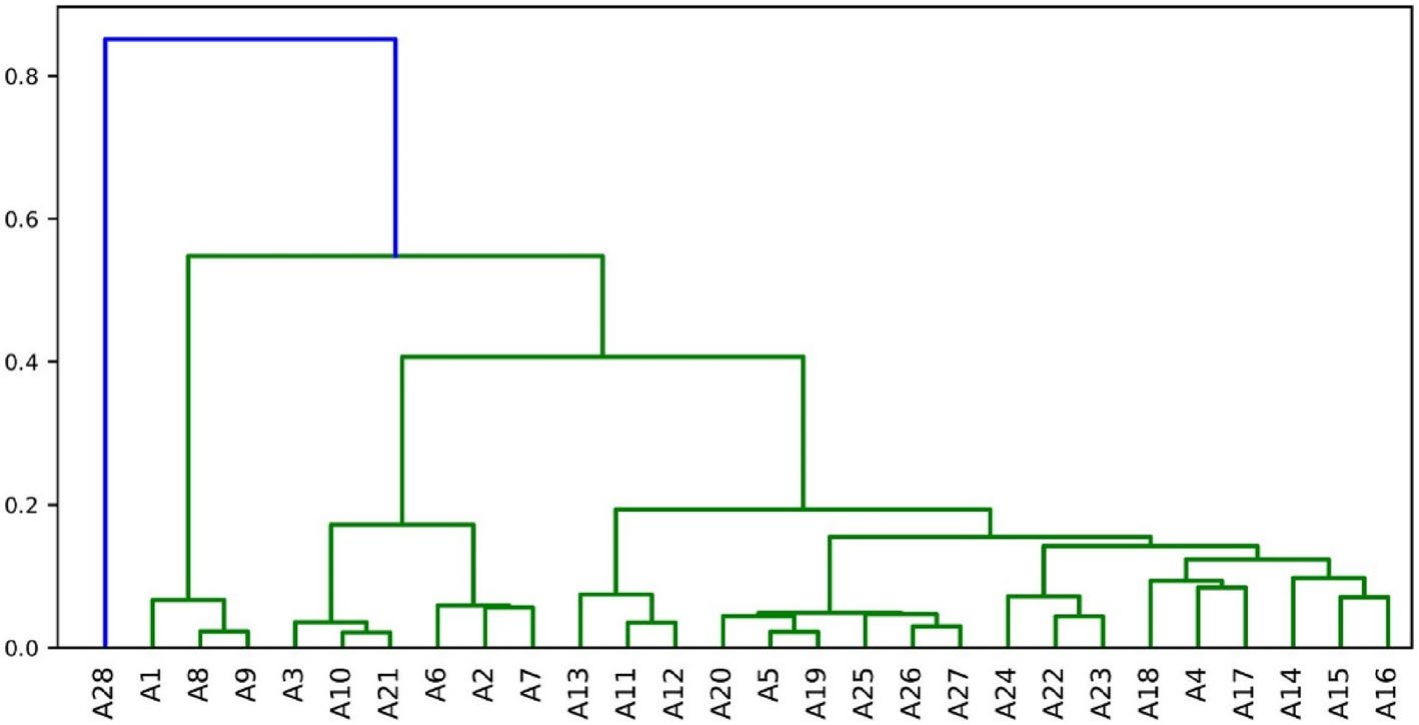
Clustering of Docking Algorithms. A hierarchical clustering of the constituent docking algorithms based on the latent features extracted by SVD (k = 5) on the AVG case.

Figure 5 illustrates the similarities between all the constituent algorithms in terms of hierarchical clustering.

At the lowest level of the clusters, the following groups of algorithms happen to be highly similar: {A8, A9}, {A10, A21}, {A2, A7}, {A11, A12}, {A5, A19}, {A26, A27}, {A22, A23}, {A14, A17}, {A15, A16}. Referring to Table ~ \ref{algorithm-configurations}, except the {A14, A17} pair, all the grouped algorithms come with the same configuration with reference to their population sizes and mutation rates. The third variation used for utilizing a different configuration at the algorithm level, the window size, does not cause any drastic changes on the behavior of those algorithms.

Regarding this aspect of algorithm similarity, by only keeping one algorithm from similar ones, a potential sub-portfolio offering comparable performance would be {A1, A2, A3, A4, A5, A6, A8, A10, A11, A13, A14, A15, A18, A20, A22, A24, A25, A26, A28}, involving 19 algorithms out of 28 options. The portfolio can be further reduced by referring to large algorithm clusters by going one level higher on the hierarchical cluster. Then, an example portfolio would be {A1, A3, A6, A13, A14, A18, A20, A24, A28}.

**Figure 6:**
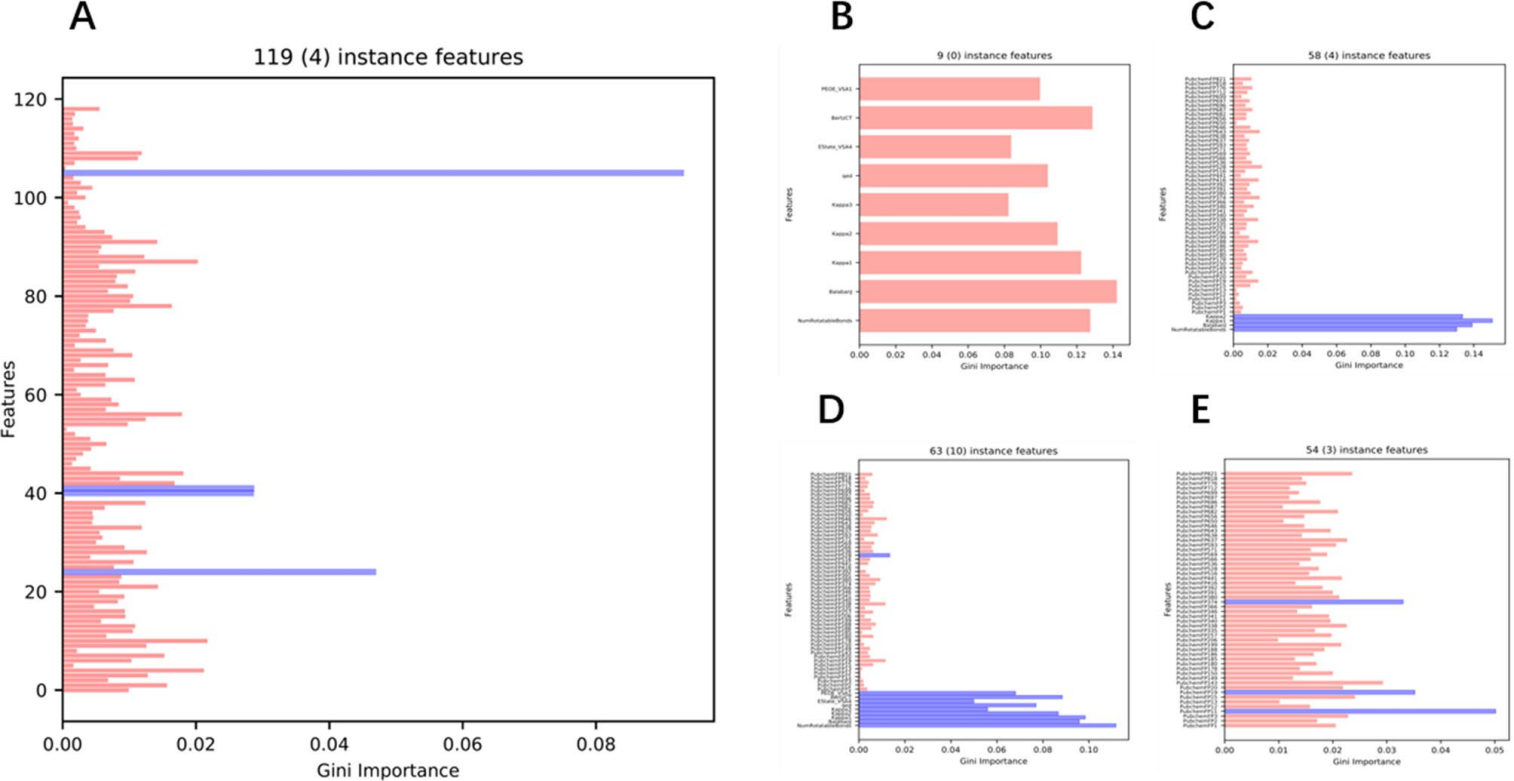
Gini Importance of Features. The blues ones are the significantly more critical than the rest concerning their Gini values. (**A**) The Gini importance values of all the docking instance features, (**B**) The Gini importance values of the $F_{md,top9}$ features, (**C**) The Gini importance values of the $F_{md,top4 + sf,top54}$ features, (**D**) The Gini importance values of the $F_{md,top9 + sf,top54}$ features, (**E**) The Gini importance values of the $F_{sf,top54}$ features.

Figure 6A visualizes the importance of the PLDP instance features. The importance aspect is determined through the Gini importance values explored while building the Random Forest (RF) prediction models under ALORS. Among these 119 features, 4 of them obtain the much higher Gini Importance, thus coming as the significantly most critical compared to the rest. The corresponding features are.

- NumRotatableBonds
- BalabanJ
- Kappa1
- Kappa2

In addition to molecular descriptors such as characteristics, $F_{md}$, substructure fingerprints, $F_{sf}$, are used to perform AS. Fingerprints are binary forms of features, each representing the presence of a highly specific sub- structure. In that respect, it is relatively hard to benefit from the individual features as in the case for molecular descriptors. Table 3 reports the ALORS’ performance with varying feature sets. The results indicate that $F_{md}$ is more informative than $F_{sf}$ as expected. Focusing on $F_{md}$, two subsets are additionally evaluated, which are $F_{md,top4}$ and $F_{md,top9}$.They are essentially the top features measured by their Gini values extracted from the original ALORS model. As mentioned above, $F_{md,top4}$ denotes the main significantly influential features, while $F_{md,top9}$ has 5 additional features besides the ones in $F_{md,top4}$ They are chosen considering the Gini importance value is cut-off from 0.15. Both subsets are good enough to outperform the standalone algorithms rather than using the complete 119 features. However, the larger subset $F_{md,top9}$ provides better results than $F_{md,top4}$. Figure 6B visualizes the contributions of each feature from $F_{md,top9}$ when an AS model is built with $F_{md,top9}$. A similar approach is followed for $F_{sf}$, resulting in a subset of 54 features, $F_{sf,top54}$. In relation to that, Fig. 6E illustrates the importance of each of these features. The use of 54 features out of 881 provided further performance improvement. Considering that the complete fingerprint feature set is rather large, an extra ALORS model is built using a higher number of tresses for RF, increasing from 100 to 500. Although superior performance with the mean rank of 6.39 5.62 is achieved compared to the default ALORS setting, the performance is still worse than the scenario using $F_{sf}$,top54. The final evaluation on the features is carried out utilizing both $F_{md}$ and $F_{sf}$, in particular their aforementioned subsets, $F_{md,top4 + sf,top54}$ and $F_{md,top9 + sf,top54}$. These combinations improved both the sole, $F_{md}$ and $F_{sf}$, feature subset based results. This outcome suggests that the substructure fingerprints come with extra information which is not directly come from the molecular descriptors. Corresponding feature importance are provided in Fig. 6C and D for $F_{md,top4 + sf,top54}$ and $F_{md,top9 + sf,top54}$ respectively.

**Table 3:** Ranking of different feature sets.

Feature set	Mean ± Sd
$F_{md}$	6.00 ± 5.14
$F_{md,top4}$	6.30 ± 5.32
$F_{md,top9}$	6.11 ± 5.16
$F_{sf}$	6.57 ± 5.83
$F_{sf,top54}$	6.29 ± 5.45
$F_{md,top4 + sf,top54}$	6.10 ± 5.20
$F_{md,top9 + sf,top54}$	08 ± 5.26

The ranking of the ALORS recommendation models with distinct instance feature sets on AVG.

### Molecular descriptor analysis

**Figure 7:**
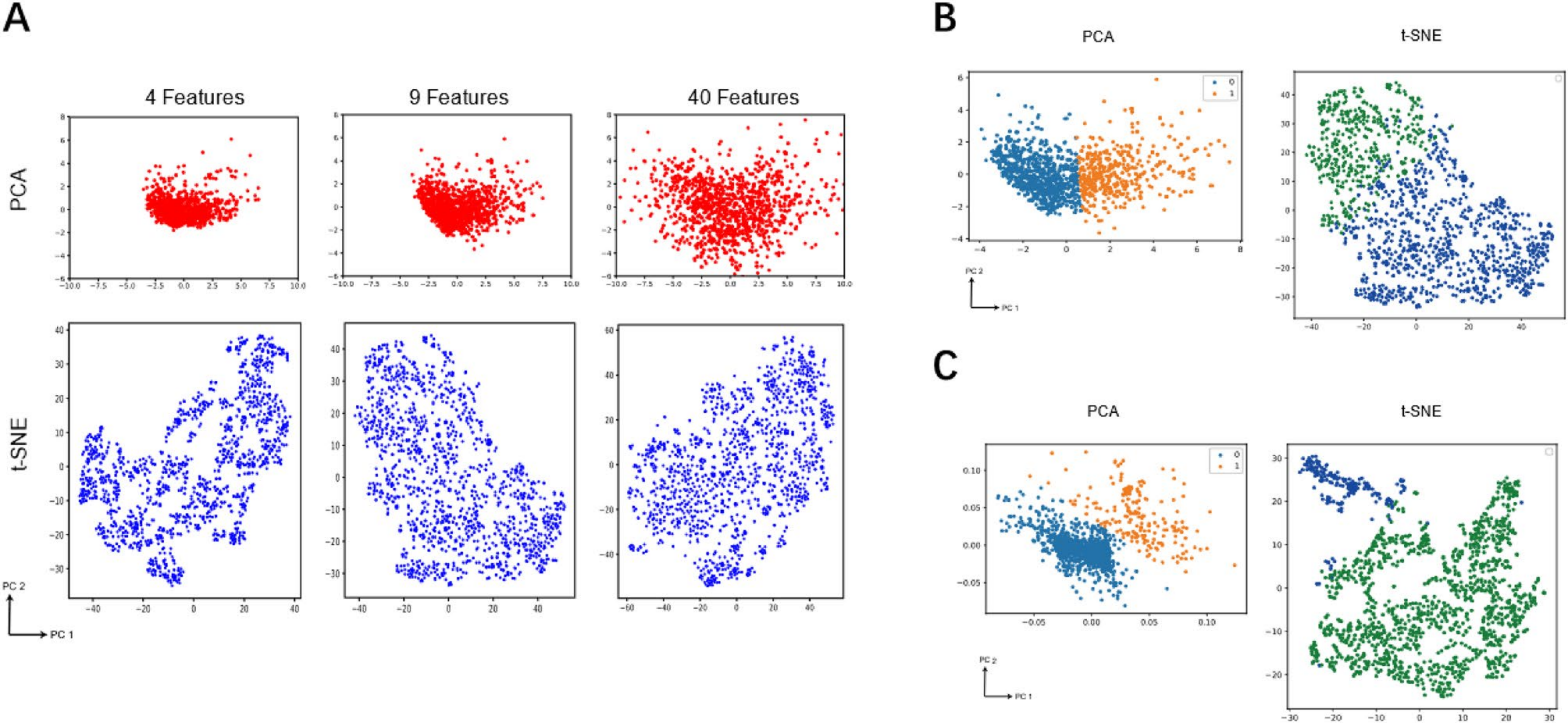
Features Visualization with PCA, t-SNE and Kmeans. (**A**) 4, 9 and 40 features visualization with PCA and t-SNE. (**B**) In 2-D PCA and t-SNE space, Kmeans classification results of 9 features. (**C**) In 2-D PCA and t-SNE space, Kmeans classification results of 5 latent features, extracted by SVD, for a different feature set.

Considering the Gini importance, top 4, top 9, and top 40 features are picked for analyzing the instance space. To visualize the instances in the 2-dimensional space, Principal Component Analysis (PCA) and t-distributed Stochastic Neighbor Embedding (t-SNE) are applied to reduce those features into 2 dimensions. The instance representations achieved by PCA and t-SNE are shown in Fig. 7A. Compared to the PCA components, t-SNE delivers more separated instance clusters. By observation and analysis, selecting the 9 features turns out to be the most discriminant. Thus, k-means algorithm^54^ is applied to cluster the instances using those 9 features. After trying different k ∈ [2, 15] values, the best k is determined as 2 with respect to the silhouette score which is derived as the mean silhouette coefficients^55^ across all the instance points.

The final results of the clustering are shown in Fig. 7B. As the score indicates, it is best to divide the 9 top features into two clusters. It is observed that there is a distinct divide in the middle of the data. While we can find a more diverse spread of points in t-SNE, the division is relatively indistinct. In PCA, where distinct groups are clustered more tightly, the clustering is clearer for the other feature set if it is divided into two groups. Also, in t-SNE, the part in the top left corner from -10 to 40 PC2 is more concentrated, whereas the other part is dispersed and sparse. Figure 7C reflects a striking situation of the second feature set where five latent features are used. The amount of data in these two clusters is distributed heterogeneously, with one group outnumbering the other to a great extent. Consequently, the pattern of a particular group can be captured.

**Figure 8:**
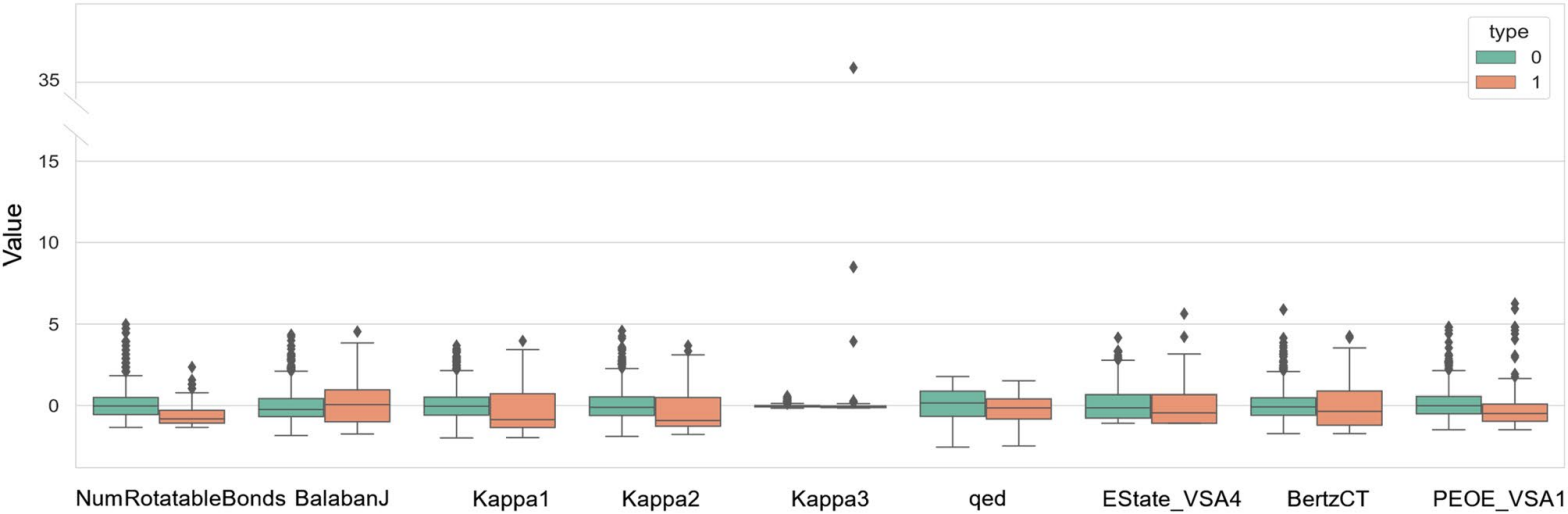
Boxplot of Features. Type 0 denote the same group 0 when conducting PCA and t-SNE and type 1 denote group 1. The distributions of 9 selected features in the two clusters are given to demonstrate the possible patterns for each group. Group 0 shows a clustered group while with more outliers compared to group 1.

It should be noted that the silhouette score cannot indicate the situation when the points are only considered as a whole group. Though we have no idea how one group performs using the evaluation of score, we can still observe that the points are actually evenly dispersed in either PCA or t-SNE. This means that it is best to consider them as a group. That is to say, there is no obvious clear division or clustered pattern when considering these features. As shown in Fig. 8, group 0 as type 0, denoted by the green color, is clustered more closely in general. Group 0 shows a higher median except for BalabanJ. Although most of the data in group 0 are clustered, there are more outliers compared to group 1. Strikingly, kappa3 shows a strange pattern where data are extremely gathered with several outliers two to three times larger than most of the data.

**Figure 9:**
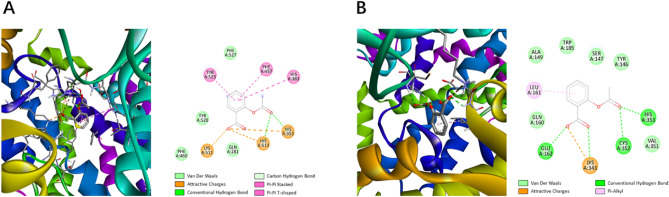
Interaction Plot of Ligand ZINC000000000053 and ACE. (**A**) under default parameter configuration, (**B**) under best parameter configuration in AutoDock4.

Figures 9A and B show the conformational and interaction difference of an instance docked with default algorithm and the best algorithm. As more hydrogen bonds are observed, the docking pose predicted by the best parameter configuration is likely to yield a more stable binding with the receptor protein compared to the pose predicted by the algorithm with default parameter configuration.

As mentioned above, using the chemical descriptors provided by the open-source python library RDKit^51^, 208 features, molecular descriptors, are generated for each of the molecules involved in the docking process. Referring to their importance, starting from the most important one, the top 9 features are (1) the number of rotatable bonds, (2) the Balaban’s J index, (3.4.5) the Kappa molecular shape index including Kappa 1,2,3, (6) the quantitative estimate of the drug-likeness index, (7) the electrotopological state index, (8) Bertz molecular complexity index, and (9) the partial equalization of orbital electronegativity index. Although these features have been highlighted by ALORS, there is an additional need to examine their applications in QSAR studies concerning whether they can be comprehended in the docking process.

### Number of bond rotations

The number of rotatable bonds can reflect the flexibility of a molecule^56^. Previous studies suggest that this.molecular descriptor helps to differentiate between drugs and other small molecules as drugs have lower flexibility^57,58^. Essentially, molecular docking is a searching process of best positions and poses under constrained docking space. Varying the number of rotatable bonds directly affects the potential docking poses returned by AutoDock. Thus, it is important to adjust the number of bond rotations, when ligands are preprocessed via AutoDock Tools^27^.

### Balaban’s J index

Balaban’s J index is one of the topological indices that treat molecules as connected graphs, which represent.the molecular structure by a single numerical number^59^. The J Index improves the discriminating power especially for isomers since it employs the average sums of distances inside the molecule. It is sensitive to the number of bonds or atoms difference. The calculation of the index is computationally efficient while preserving the physical and structural information of the molecule^60,61^.

### Kappa 1/2/3

The Kappa molecular shape index is another type of topological index that focuses on molecular shape information. The kappa molecular shape index quantifies the difference between the most complex and the potentially simplest conformation^62^. Kappa 1, 2, and 3 are able to discriminate between isomers that cannot be distinguished if measured by the number of atoms or bonds^63^. Therefore, kappa molecular shape indices are reliable descriptors for measuring the overall connectivity of a molecule.

### QED

QED is short for quantitative estimation of drug-likeness, which was proposed to provide a practical guidance in drug selection as a refined alternative to Lipinski's rule of five^64^. QED is an integrated index that comprises 8 physical properties of molecules, including the octanol–water partition coefficient, the number of hydrogen bond donors and acceptors, the molecular polar surface area, the number of rotatable bonds, the number of aromatic rings and the number of structural alerts. QED has been applied in virtual screening of large compound databases to filter favorable molecules^65^ and to aid the building and benchmarking of deep learning models for de novo drug design^66^. QED’s strength is also mirrored by the given Gini importance.

### EState_VSA4

EState_VSA descriptor compromises both EState (electrotopological state) and the VSA index. EState index contains atom-level and molecular level topology information^67^. Unlike the Kappa molecular shape index, which emphasizes the structure of molecules, the electrotopological state index reveals the electronegativity of each atom as well as the weighted electronic effect. It has been validated by its strong correlation with the 17O NMR shift in ethers and the binding affinity of various ligands^68,69^. VSA is the Van der Waals surface area value of an atom, and it is used to determine whether EState indices are calculated. Regarding molecular docking, the electrostatic interaction between the ligand and the receptor is a significant component of the energy evaluation in AutoDock’s semi-empirical force field computation, which may explain why it ranks eighth out of 208 descriptors.

### BertzCT

Bertz index was defined to represent the complexity of a molecule quantitatively derived from molecular graphs^70^. It comprises two properties of the molecule: the number of lines in the line graph and the number of heteroatoms. As both heterogeneity and connectivity are integrated into one index, abundant information is extracted from the molecule. BertzCT is particularly useful in organic synthesis. It can be used to monitor the complexity of synthetic products, and thus evaluate intended synthesis route prior to the implementation^71^.

### PEOE_VSA1

PEOE_VSA is another hybrid descriptor consisting of the partial equalization of orbital electronegativity and the Van der Waals surface area. The partial equalization of orbital electronegativity (PEOE) was first presented to assess reactivity in chemical synthetic design^72^. PEOE obtains the partial charges based on the atomic orbital electronegativity iteratively throughout the entire molecule. The electronegativity of atoms can be accurately computed in complex organic molecules even with electron withdrawing and donating effects. PEOE was first tested to model the taste of compounds and later applied to QSAR studies that included prediction of anesthetic activity and inhibition of HIV integrase^73,74^. To simulate in vivo environment, it is highly suggested to assign partial charges to ligands to obtain a reliable binding energy in AutoDock.

## Conclusion

This paper is aimed at introducing and further evaluating ALORS as a recommender system-based algorithm selection system which automatically selects LGA variants on a per-instance basis on AutoDock. Features that include molecular descriptors and fingerprints pertaining to each protein–ligand docking instance have been employed to quantify chemical compounds. The study has shown that ALORS delivers the best results compared to all candidate algorithms from a fixed algorithm pool. Nine features have been highlighted as significant determinants of the protein–ligand interaction and are analyzed to inspire exploration into chemical features that are critical to docking performance. The findings of this research accentuate utilizing a suitable algorithm selector and features to best approach a molecular docking task that searches for druggable compounds. ALORS has the potential to become the preferred choice for performing protein–ligand docking tasks for CADD research. What’s more, the results of our study add to the rapidly expanding applications of automatic algorithm selections.

However, one limitation of our study is that ACE was the only protein adopted for the docking data generation. Although ALORS works well in the docking case with ACE; nevertheless, the generalizability of our model to other proteins remains to be determined. More proteins should be incorporated to our model to increase the diversity of protein–ligand interaction. Therefore, extending the docking scenarios with varied target proteins may present a more comprehensive evaluation of the performance of ALORS as an AS tool. At the same time, hand-selected characteristics of molecules derived from empirical evidence are equally viable options. Hand-selected features that are more specific and relevant can be mixed with algorithm-selected features to achieve more relevance and precision.

Other protein–ligand docking programs such as DOCK, Glide, and CABSdock are also recommended, and the underlying algorithm of each docking platform may be tailored to specific docking situations. AutoDock performs well in automated ligand docking to macromolecules because of its improved LGA search algorithm and empirical binding-free scoring function, but it remains to be seen whether exhaustive search-based docking programs such as Glide and DOCK that use the Geometric Matching Algorithm perform better in other areas. Further focus can be directed towards evaluating and automatically selecting the best docking programs in different docking scenarios.

During the study, we noticed the increasing prevalence of the application of Neural networks (NN) in protein–ligand interaction prediction. Neural networks, which are composed of layers and neurons to recognize patterns such as numerical vectors, images, texts, sounds, and even time series, are widely used for classification or prediction tasks. Under the frame of Neural networks, Graph neural networks (GNNs) rely on characterizing data as graphs that consist of nodes and edges and excel in capturing the nonlinear relation in images compared with traditional regression or classification models^75^. GNNs are particularly useful for graph data that have relational information. As molecules are bonded structures, natural information for chemicals can be represented as irregular molecular graphs. The image-based features derived from molecules bring about more promising results than the traditional characteristics derived from molecular descriptors^76^. Consequently, more efforts can be put into the implementation of GNNs for better prediction of protein–ligand interaction.

## Supplementary Information

Supplementary Information.

## Data Availability

The receptor, ACE, can be found with PDB DOI: 1O86, and docking ligands are in ZINC15 database: https://zinc15.docking.org/catalogs/dbfda/.

## Abbreviations

ACE: Human angiotensin-converting enzyme
LGA: Lamarckian-genetic algorithm
ALORS: Algorithm recommender system
DD: Drug discovery/design
CADD: Computer-aid drug discovery/design
SARs: Structure activity relationships
QSARs: Quantitative structure activity relationships
AS: Algorithm selection
PLDP: Protein-ligand docking problem
NFLT: No free lunch theorem
GA: Genetic algorithm
LS: Local search
CF: Collaborative filtering
MF: Matrix factorization
SVD: Singular value decomposition
RF: Random forest
FDA: Food and drug administration
MOL: Molecular data file
PDB: Protein data bank
PDBQT: Protein data bank, partial charge (Q), & atom type (T)
AVG: Average
PCA: Principal component analysis
t-SNE: T-distributed Stochastic neighbor embedding
QED: Quantitative estimation of drug-likeness
PEOE: Partial equalization of orbital electronegativity
HIV: Human immunodeficiency virus
NNs: Neural networks
GNNs: Graph neural networks
