# Supplementary material for: Algorithm selection for protein–ligand docking: strategies and analysis on ACE
Source: Sci Rep. 2023 May 22;13:8219. doi: 10.1038/s41598-023-35132-5 (PMC10201035; doi:10.1038/s41598-023-35132-5)
Supplement: Supplementary file 1 — Supplementary Information. [file 41598_2023_35132_MOESM1_ESM.docx]

**Algorithm Selection for Protein-Ligand Docking: Strategies and Analysis on ACE: Supplementary Information**

Tianlai Chen,^1^ Xiwen Shu,^1^ Huiyuan Zhou,^1^ Floyd A. Beckford^1, †^ and Mustafa Misir,^1, †^

1. Division of Natural and Applied Sciences, Duke Kunshan University

^†^Corresponding authors: [floyd.beckford@duke.edu](mailto:floyd.beckford@duke.edu) and mustafa.misir@dukekunshan.edu.cn


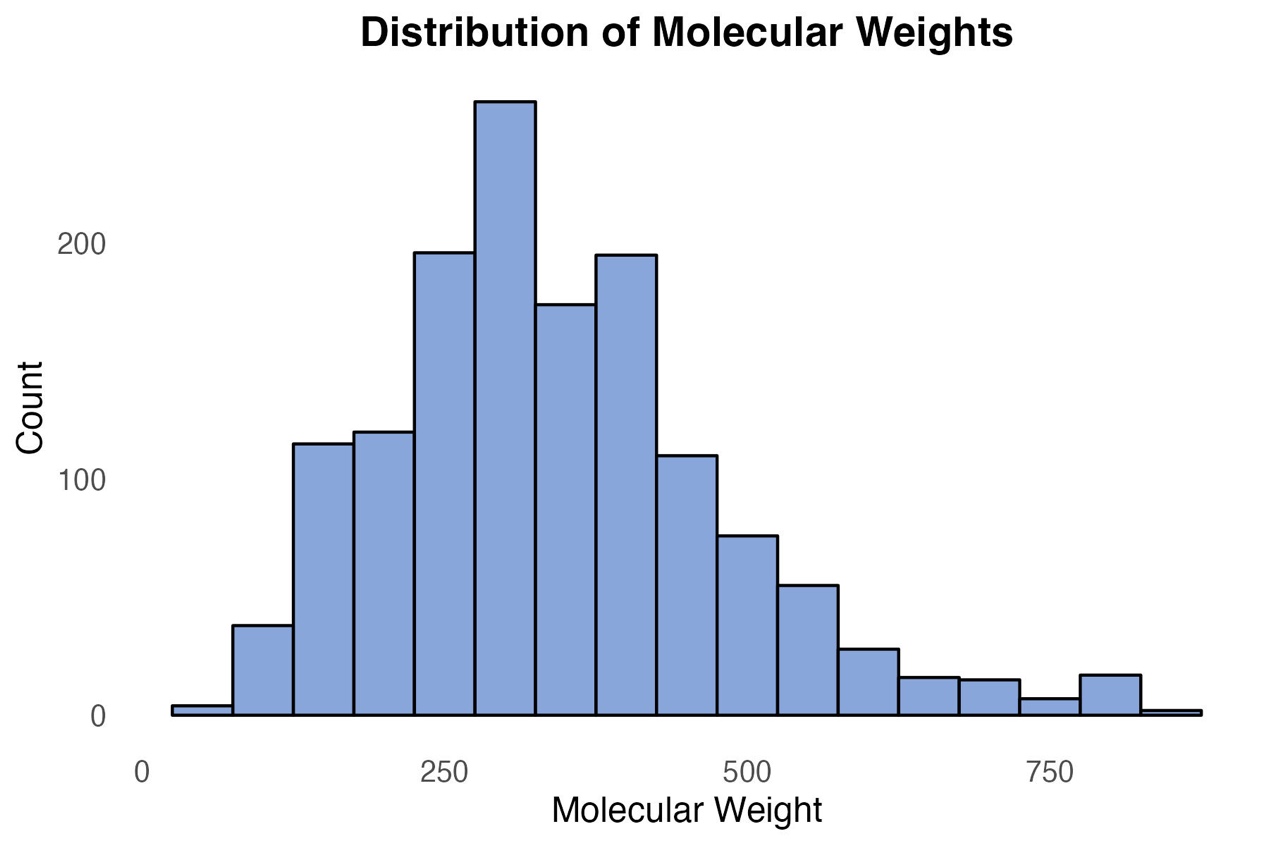


**Supplementary Figure 1.** Distribution of molecular weights

**
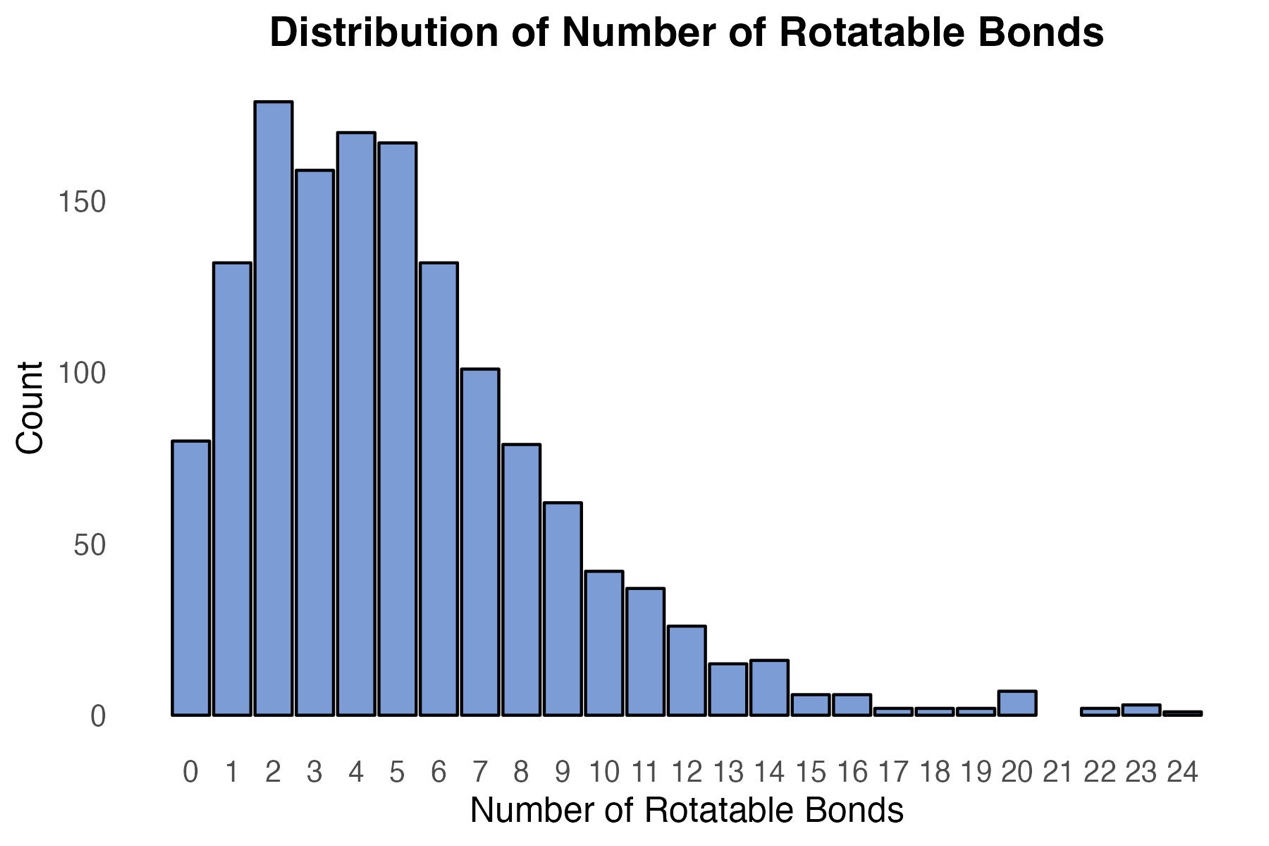
**

**Supplementary Figure 2.** Distribution of the number of rotatable bonds

**
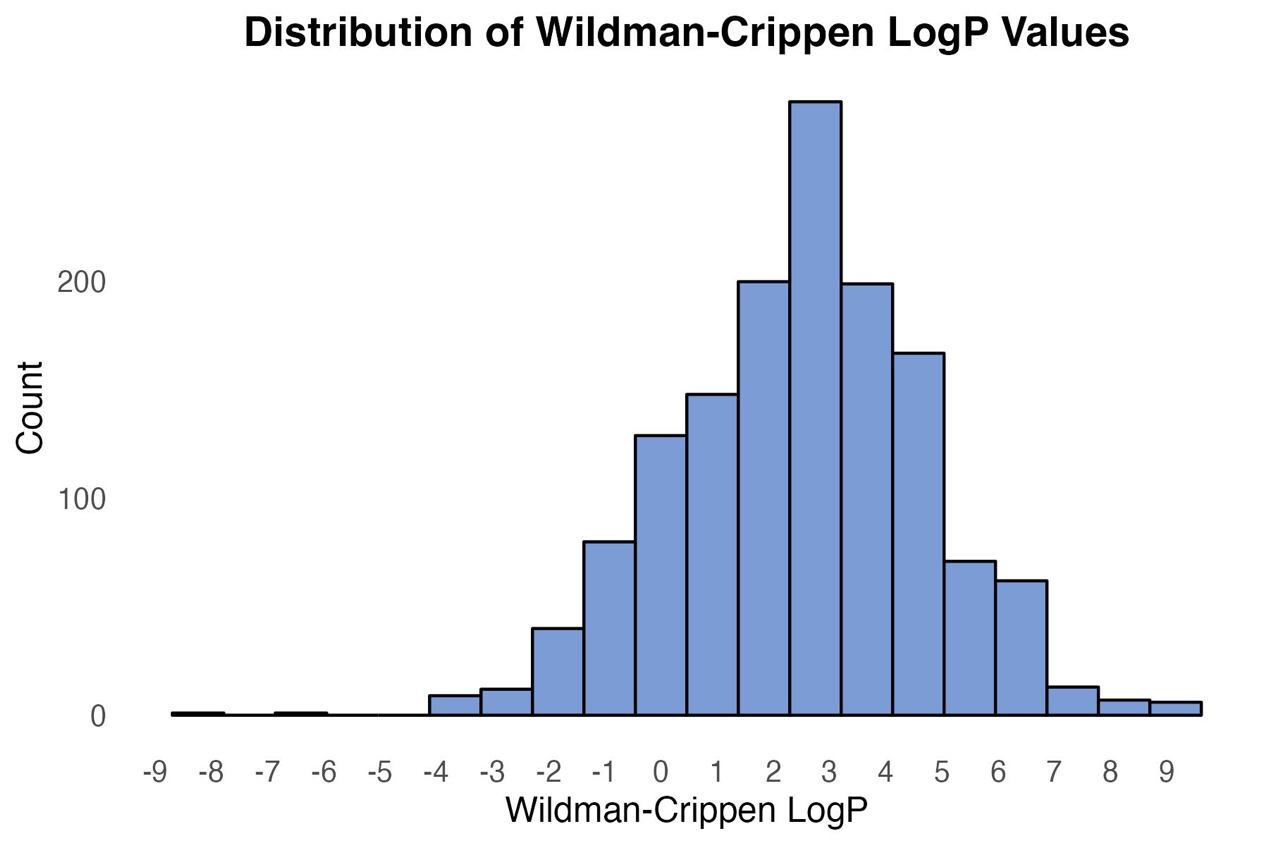
**

**Supplementary Figure 3.** Distribution of the Wildman-Crippen LogP values

**
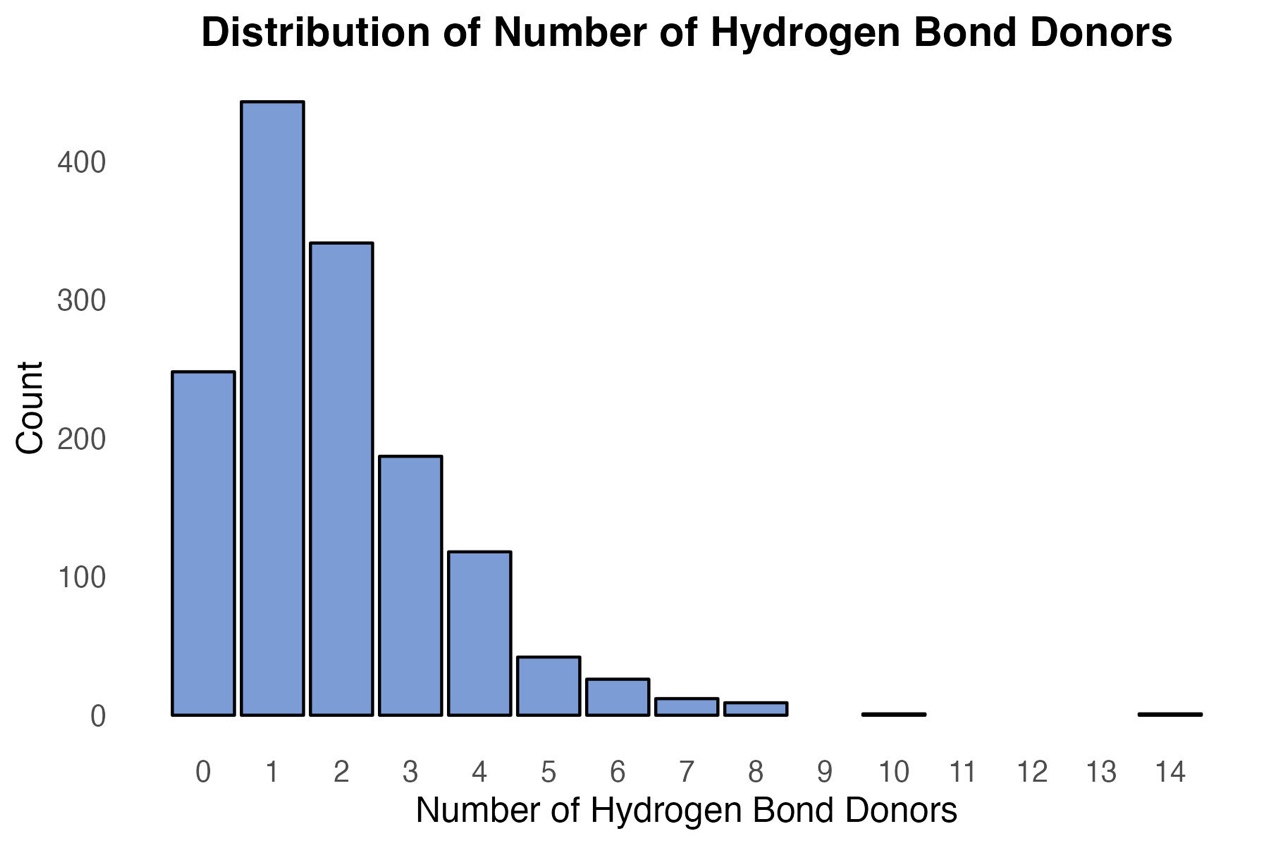
**

**Supplementary Figure 4.** Distribution of the number of hydrogen bond donors

**
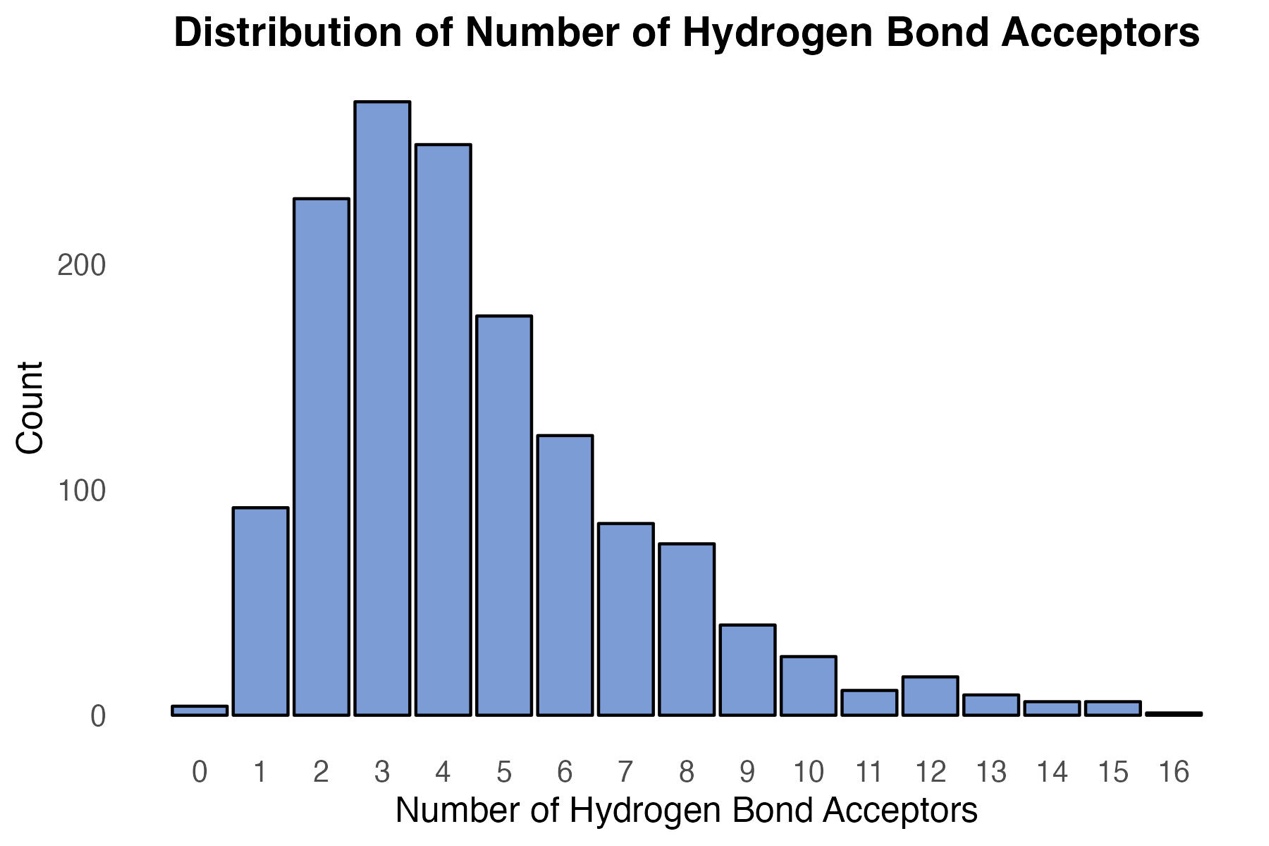
**

**Supplementary Figure 5.** Distribution of the number of hydrogen bond acceptors
